# Erratum for Chou et al., “Novel Cytomegalovirus UL54 DNA Polymerase Gene Mutations Selected *In Vitro* That Confer Brincidofovir Resistance”

**DOI:** 10.1128/aac.01024-26

**Published:** 2026-08-07

**Authors:** Sunwen Chou, Ronald J. Ercolani, E. Randall Lanier

## ERRATUM

Volume 60, no. 6, p. 3845-3848, 2016, https://doi.org/10.1128/aac.00214-16. Table 1: A production error resulted in the EC_50_ concentration units for the drugs Cidofovir, Ganciclovir, and Foscarnet being incorrectly displayed as “nM.” The concentration units should read “µM” as submitted.

